# All-suture anchor pullout results in decreased bone damage and depends on cortical thickness

**DOI:** 10.1007/s00167-020-06004-6

**Published:** 2020-04-24

**Authors:** Dimitris Ntalos, G. Huber, K. Sellenschloh, H. Saito, K. Püschel, M. M. Morlock, K. H. Frosch, T. O. Klatte

**Affiliations:** 1 Department of Trauma and Orthopaedic Surgery, University Medical Center Hamburg-Eppendorf, Martinistraße 52, 20246 Hamburg, Germany; 2 Institute of Biomechanics, TUHH Hamburg University of Technology, Hamburg, Germany; 3 Molecular Skeletal Biology Laboratory, University Medical Center Hamburg-Eppendorf, Hamburg, Germany; 4 Institute of Forensic Medicine, University Medical Center Hamburg-Eppendorf, Hamburg, Germany

**Keywords:** Shoulder surgery, Arthroscopy, Rotator cuff, Suture anchor, All suture, Pullout, Bone mass density, Cortical thickness, Micro-CT

## Abstract

**Purpose:**

To evaluate the influence of cortical and cancellous bone structure on the biomechanical properties of all-suture and conventional anchors and compare the morphological bone damage after their failure. The hypothesis of the study is that all-suture anchor pullout is less invasive and that the pullout force is influenced by the cortical thickness.

**Methods:**

Thirty human humeri were biomechanically tested as follows: starting with a load cycle from 20 to 50 N, a stepwise increase of the upper peak force by 0.05 N for each cycle at a rate of 1 Hz was performed. Analysis included maximum pullout strength for three different anchor implantation angles (45°, 90°, 110°) of the two anchor types. After anchor pullout, every sample underwent micro-CT analysis. Bone mineral density (BMD) and cortical thickness were determined at the anchor implantation site. Furthermore, the diameter of the cortical defect and the volume of the bone cavity were identified.

**Results:**

The maximum pullout strength of all-suture anchors demonstrates a strong correlation to the adjacent cortical thickness (*r* = 0.82, *p* ≤ 0.05) with at least 0.4 mm needed to withstand 200 N. No correlation could be seen in conventional anchors. Moreover, no correlation could be detected for local BMD in both anchors. All-suture anchors show a significantly narrower cortical defect as well as a smaller bone cavity following pullout (4.3 ± 1.3 mm vs. 5.3 ± 0.9 mm, *p* = 0.037; 141 mm^3^ vs. 212 mm^3^; *p* = 0.009). The cortical defect is largest if the anchors are placed at a 45° angle.

**Conclusion:**

In contrast to conventional anchors, the pullout force of all-suture anchors depends on the thickness of the humeral cortex. Furthermore, all-suture anchors show a significantly smaller cortical defect as well as decreased bone damage in the case of pullout. Therefore, the clinical implication of this study is that all-suture anchors are advantageous due to their bone preserving ability. Also, intraoperative decortication should not be performed and cortical thickness should be preoperatively evaluated to decrease the risk of anchor failure.

## Introduction

All-suture anchors (ASA) are increasingly used in arthroscopic rotator cuff repair and are entirely composed of suture material [2, 4, 20, 22]. The bone–anchor interface is constituted by anchor deployment against the inner cortical wall of the bore hole through pulling by the surgeon. During this process, the diameter of the all-suture anchor increases while the initial construct length is reduced [3, 10, 11]. In contrast to conventional suture anchors (CA), this type of fixation is thought to be bone-preserving due to the removal of less bone during insertion or pullout [3, 10, 19]. Moreover, ASA have smaller footprints enabling more points of fixation in the same region, as well as maintaining substrate for future potential surgeries or in case of revision [10, 11, 20, 27]. There are several biomechanical studies comparing all-suture to conventional anchors. The results of these studies demonstrate similar or slightly inferior results regarding maximum pullout strength or gap formation for all-suture anchors [11, 20, 27]. Decortication of the footprint prior to suture anchor insertion to potentially increase the healing potential at the bone–tendon interface is controversial and might adversely affect load to failure of suture anchors, especially ASA [18, 24, 27]. In contrast to conventional suture anchors, ASA are thought to be more dependent on the cortical bone than the cancellous bone where they are inserted for fixation [11].

Still, the different interactions with the cortical and cancellous bone in all-suture and conventional suture anchors are rarely described. Therefore, the aim of the study was to compare the morphological bone damage of all-suture and conventional suture anchors after pullout and to identify how the cortical and cancellous bone structure influences the biomechanical properties of the two different anchor types. The hypothesis of the study is that all-suture anchor pullout is less invasive and that the pullout force is influenced by the cortical thickness.

## Materials and methods

Approval by the institutional review board was obtained (Ethical Review Committee Hamburg, Germany; study number: WF-27/17). A total of thirty human humeral bones (15 matched pairs) were collected from donors aged between 50 and 73 years. The donors’ consent to the postmortem tissue donation was given in written form prior to death and/or by the next of kin of the deceased person. After harvesting, the specimens were sealed in plastic bags and stored at a temperature below − 20 °C. At the day of testing, each thawed humerus was perpendicularly transected at the mid-diaphyseal level and all overlaying soft tissue, including the rotator cuff, was dissected to expose the bone surface of the greater tubercle, the junction of the greater tubercle and the humeral head articular surface. To secure an intact cortex at the insertion site, no further preparation of the tuberosity was performed. The specimen was potted upright in a steel tube and the distal end was fixed using a Methyl methacrylate solution (Technovit 4004, Heraeus Kulzer, Hanau, Germany). Fixation was performed up to 2 cm proximal to the lower humeral bone–cartilage junction. Throughout preparation and testing, the specimens were wrapped in moist tissue to preserve the constitution of the tissue. The experiments were performed at room temperature (20–22 °C) in a normal room environment. Osteoporotic samples with a vBMD < 80 mg/cm^3^ were excluded.

Two different anchor types were tested in matched humeri: a commercially available all-suture anchor (ASA) with a drill size of 2.8 mm and a diameter exceeding 5 mm after unrestricted dilatation (Y-Knot RC^®^, ConMed, New York, NY) and a conventional 4.50 mm PEEK (polyetheretherketone) Suture Anchor (CrossFT™, ConMed, New York, NY). These anchors were chosen due to their comparable diameter after anchor implantation. Anchor placement was performed within the proximal anterior part of the greater tubercle 1 cm posterior of the bicipital groove, as previously described [15, 20, 29]. For the ASA, the second of the different circumferential laser marks on the inserter was chosen assuring a similar anchor depth in each sample (25 mm depth). Anchor implantation was performed according to the manufacturer’s instructions at three predefined angles (45°, 90° and 110°) relative to the bone surface. These values were chosen since they represent the range from 45° to the most obtuse angle. The previously published angle of 135° [6] was not attainable due to the anatomical impediment of the acromion [6]. Five humeral heads were tested per anchor type at each angle. After anchor insertion, the emerging suture threads were wrapped around a pulley and secured with clamps. To ensure an exact angle of insertion in each sample, two guiding platforms with a defined angle of 45° and 110° were used. The humeral heads were perpendicularly fixed to the chosen platform so that every anchor could be inserted vertically at the same predefined angle. Before the inserter was removed, the insertion angle was cross-checked with a goniometer measuring the angle between the bone surface and the inserter of the anchor. A clamp to anchor distance of 10 cm was chosen, as previously published [20]. Since the load was not applied by the cuff muscle but by the threads aligned in their loading direction, the humeral head was protected by a plastic cover. This avoided the previously reported non-physiological suture failure by cutting through the bone [25]. Biomechanical testing was performed using a servo-hydraulic testing machine (MTS Bionix 858.2, MTS Systems, Eden Praire, MN).

The loading protocol was designed to simulate the rehabilitation phase after rotator cuff repair as follows: starting with an initial load cycle from 20 to 50 N, a stepwise increase in the upper peak force by 0.05 N for each cycle at a rate of 1 Hz was performed (e.g. after 100 cycles the load ranged from 20 to 55 N) [21, 22]. Cyclic extension was continued until system failure. Recorded data for each pull out test included the number of completed cycles and the maximum pullout force. Two samples (2/30)—one with an all-suture and one with a conventional anchor—had to be excluded from the analysis since technical failure occurred rendering the specimen unusable for the experiments. Video analysis was performed throughout experiments to ensure an accurate follow through at every time point.

**Fig. 1:**
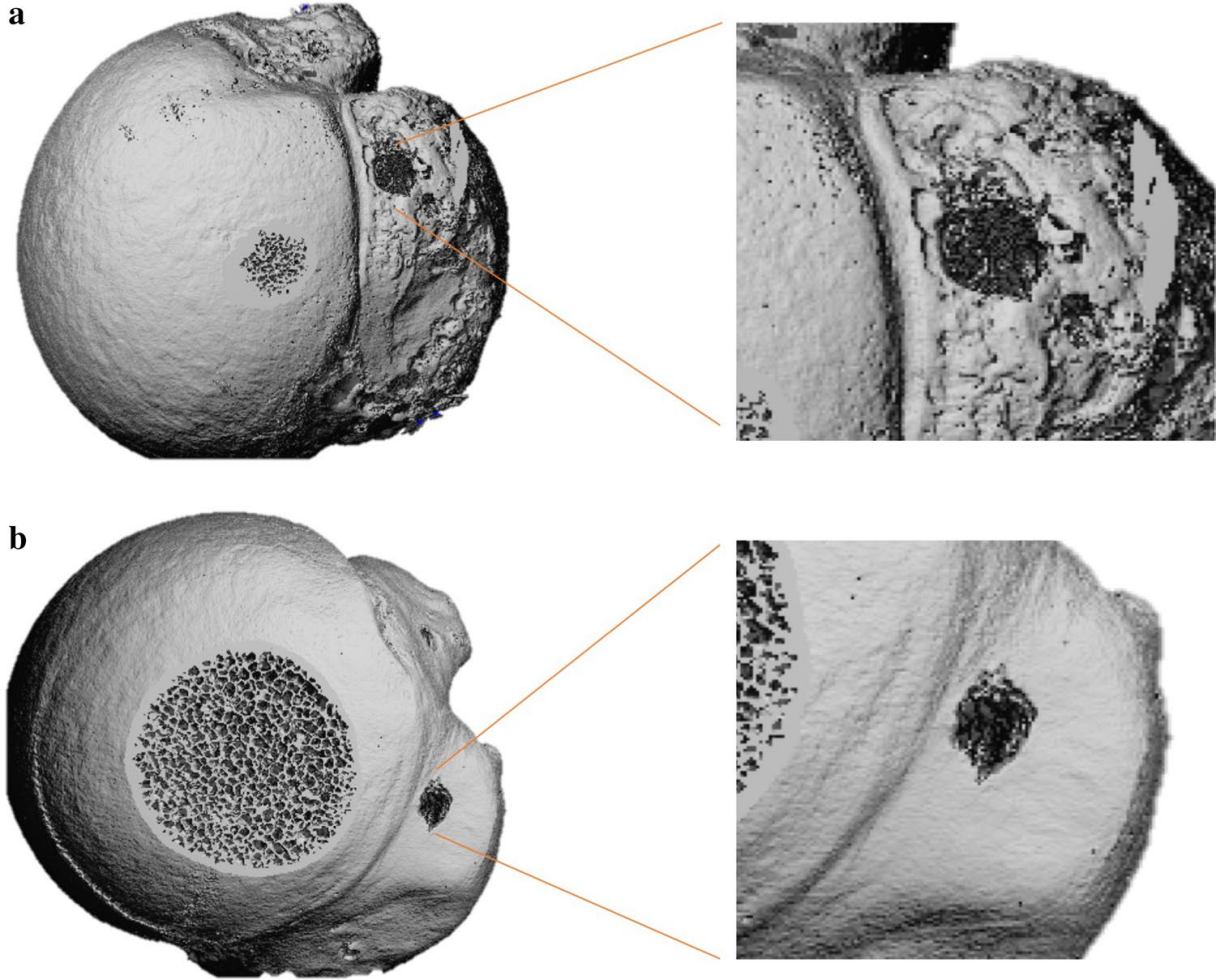
Micro-CT imaging of cortical defect following **a** conventional anchor and **b** all-suture anchor pullout

**Fig. 2:**
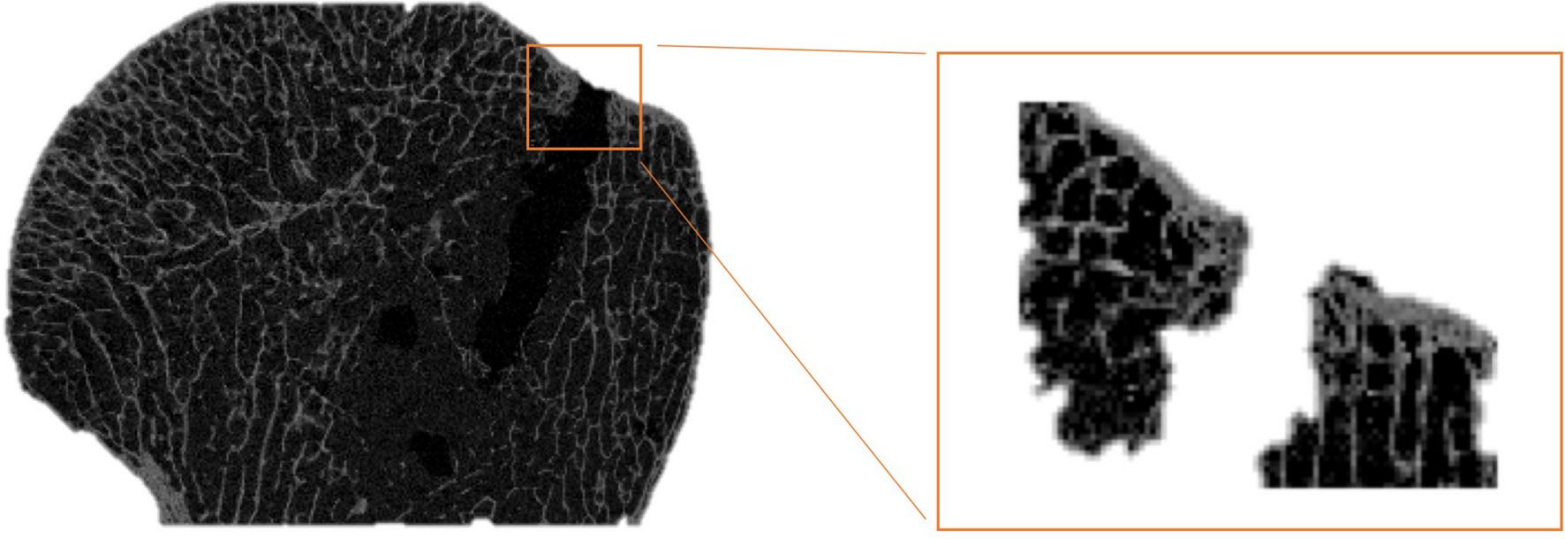
Micro-CT imaging showing sagittal plain to determine cortical thickness adjacent to the pullout hole

**Fig. 3:**
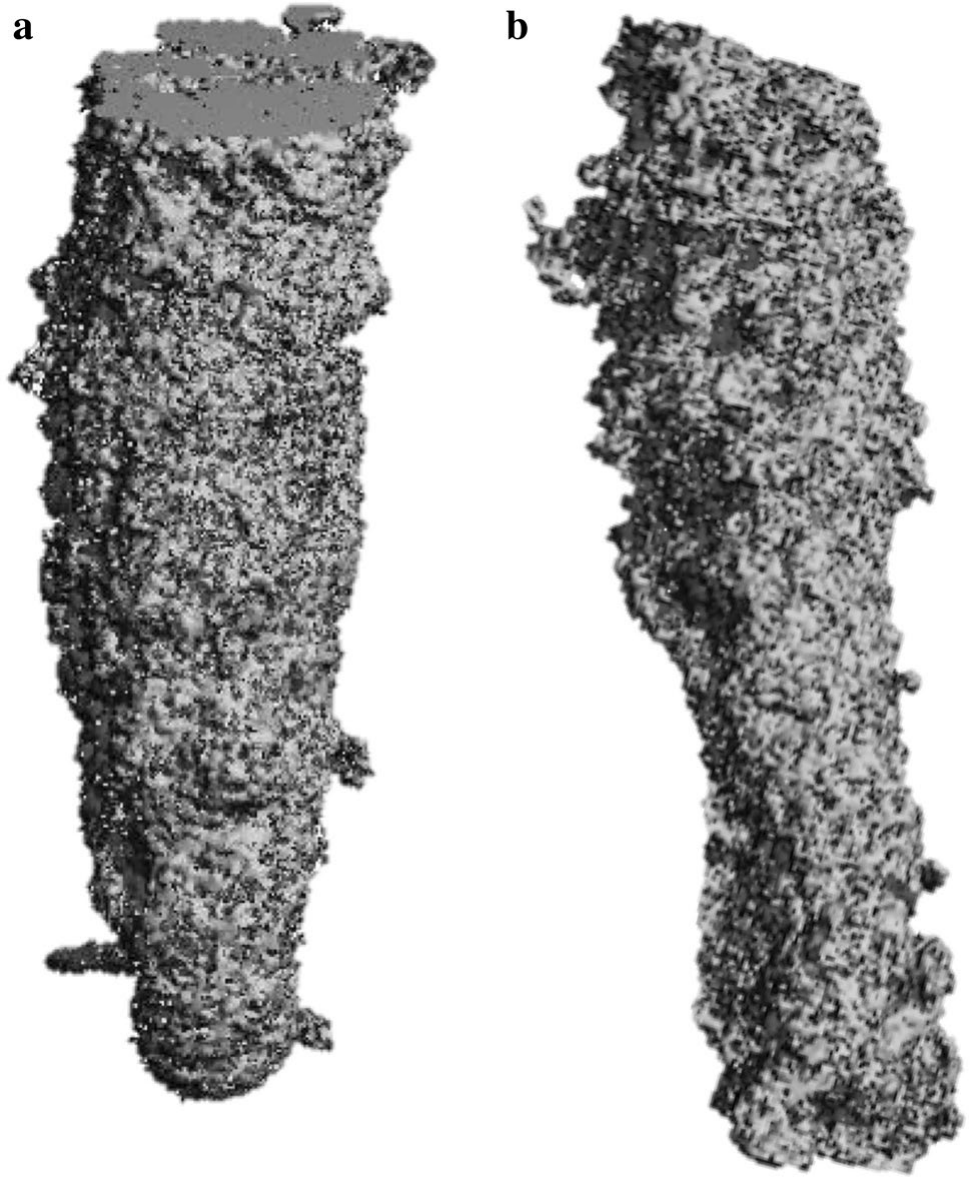
3D reconstruction through micro CT imaging of the bone cavity after **a** conventional anchor and **b** all-suture anchor pullout

**Fig. 4:**
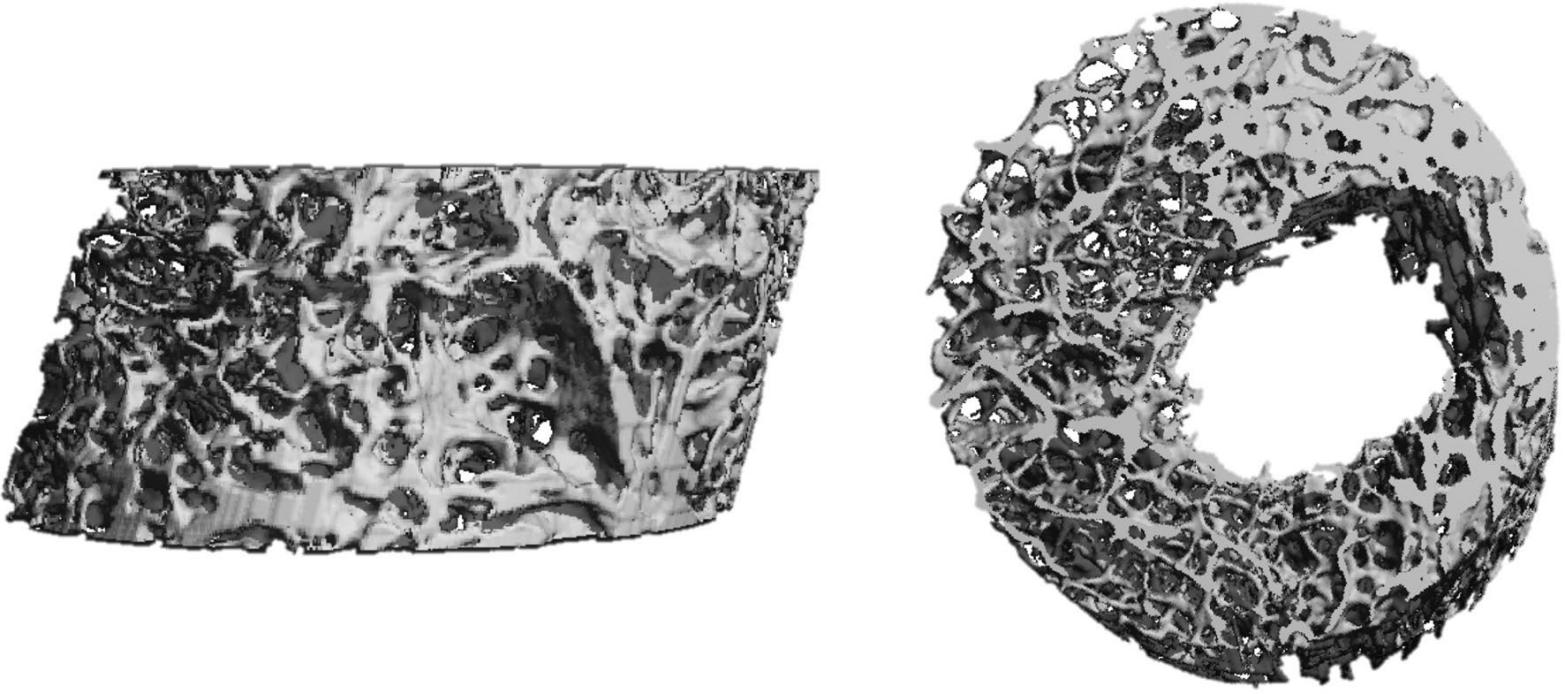
3D reconstruction through micro-CT imaging to determine bone mass density of the cancellous bone at the site of pullout

Following anchor pullout, each sample underwent micro-CT analysis (VivaCT 80, 70 kV, 114 μA, 400 ms, Fa. Scanco Medical, Wangen-Brüttisellen, Switzerland). After automatic 3D reconstruction of the humeral head (VivaCT evaluation program V6.6, Fa. Scanco Medical, Wangen-Brüttisellen, Switzerland), the diameter of the pullout hole was measured in each sample. Therefore, the illustration was oriented in the transversal plane of the cortical defect and the respective maximum diameter was measured (Fig. 1). The cortical thickness was determined in the centre of the pullout hole (oriented in the sagittal plane) and directly measured using the measurement tool of the VivaCT evaluation program V6.6 (Fig. 2). The volume of the pullout cavity was calculated using a cylindrical shape enveloping the cavity in the transversal plain (Fig. 3). Furthermore, the volumetric bone mineral density of the cancellous bone (vBMD in terms of mgCaHA per cm^3^) was determined in the region of interest (ROI) with the ROI being defined as a standardized cylindrical body surrounding the pullout defect (Fig. 4). Distances are given with the accuracy of one decimal place according to the VivaCT evaluation program—volumes are presented in mm.

### Statistical analysis

Analyses of variance with the factors insertion angle and anchor type were performed using SPSS (Version 21, IBM, Armonk, USA) with a significance level of *α* = 0.05. Normal distribution was tested using Kolmogorov–Smirnov analysis. Potential correlation between BMD, cortical thickness and pullout strength was displayed by calculating the Pearson correlation coefficient through linear regression analysis. In vitro experiments are always limited by the numbers of specimens available. To account for this, non-significant findings (n.s.) were given together with the corresponding power to allow appraisal of the used sample size.

## Results

The mean age of the 28 human cadaveric samples that underwent micro-CT analysis following anchor pullout was 61 years (SD ± 11 years).

Measurements of the cortical defect after anchor pullout showed an almost 20% smaller defect induced by all-suture anchors compared to conventional anchors. Disregarding the implantation angle, all-suture anchors (*n* = 14) showed a mean pullout diameter of 4.4 ± 1.2 mm whereas the cortical defect following conventional anchor pullout (*n* = 14) showed a mean diameter of 5.4 ± 1.0 mm (*p* = 0.034). Regarding the size of cortical defect, the two-factor model showed a significant influence of the insertion angle (*p* = 0.001) as well as the anchor type (*p* = 0.013), but not the interaction (n.s., power 0.266). Anchor insertion at an angle of 45° resulted in increased cortical damage in both anchor types compared to 90° (*p* = 0.002) and 110° (*p* = 0.021) (Fig. 1, Table 1). The cortical defect after 45° insertion ranged from 5.0 to 6.3 mm in ASA and 4.4 to 7.5 mm in CA. Anchor insertion at an angle of 90° and 110° resulted in a cortical hole, ranging from 2.8 to 4.0 mm and 3.8 to 6.5 mm (ASA) and 4.5 mm to 5.7 mm and 3.8 to 5.0 mm (CA).

**Table 1:** Mean cortical diameter and cancellous volume of the pullout hole in each anchor type

Angle of insertion	Diameter cortical defect ASA (mm)	Diameter cortical defect CA (mm)	Volume bone cavity ASA (mm^3^)	Volume bone cavity CA (mm^3^)
45°	5.5 ± 0.8	6.3 ± 1.4	136 ± 22	202 ± 24
90°	3.4 ± 0.5	5.0 ± 0.4	115 ± 18	237 ± 135
110°	4.5 ± 1.4	4.7 ± 0.6	179 ± 50	193 ± 66
Mean (*n* = 14)	4.4 ± 1.2	5.4 ± 1.0	141 ± 39	212 ± 88

Measurement of cortical thickness adjacent to the place of anchor insertion revealed a mean cortical thickness of 0.6 ± 0.3 mm (*n* = 28). The mean BMD of the cancellous bone surrounding the pullout hole was 105 ± 35 mg HA/cm^3^ (*n* = 28).

**Fig. 5:**
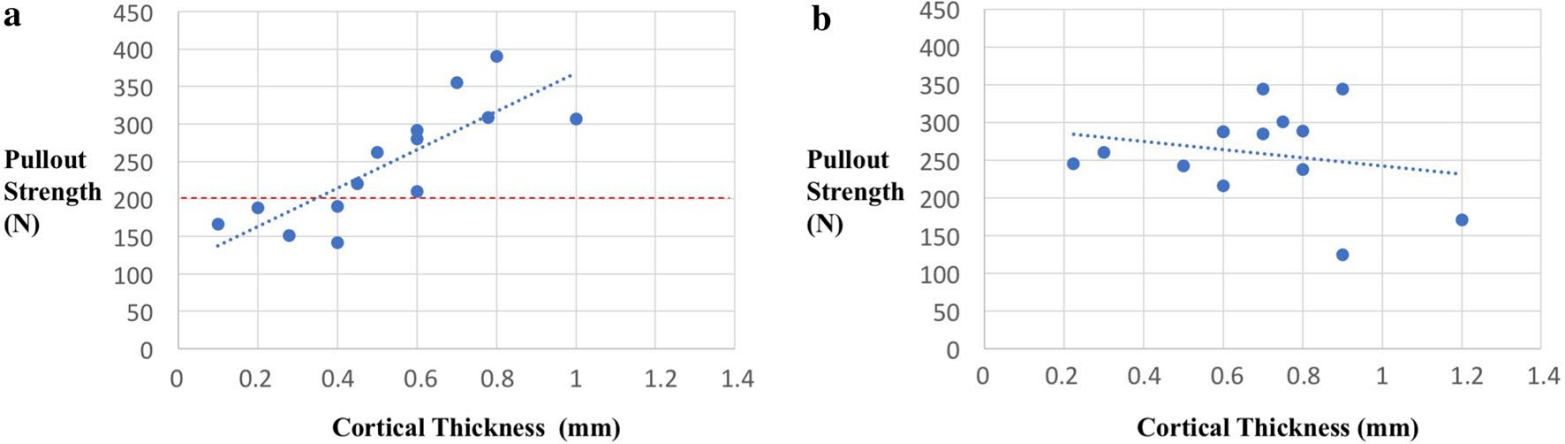
Correlation of **a** all-suture anchor and **b** conventional anchor pullout strength to cortical thickness. The red line indicates the previously estimated maximum force of 200 N which suture anchors may need to withstand in vivo

**Fig. 6:**
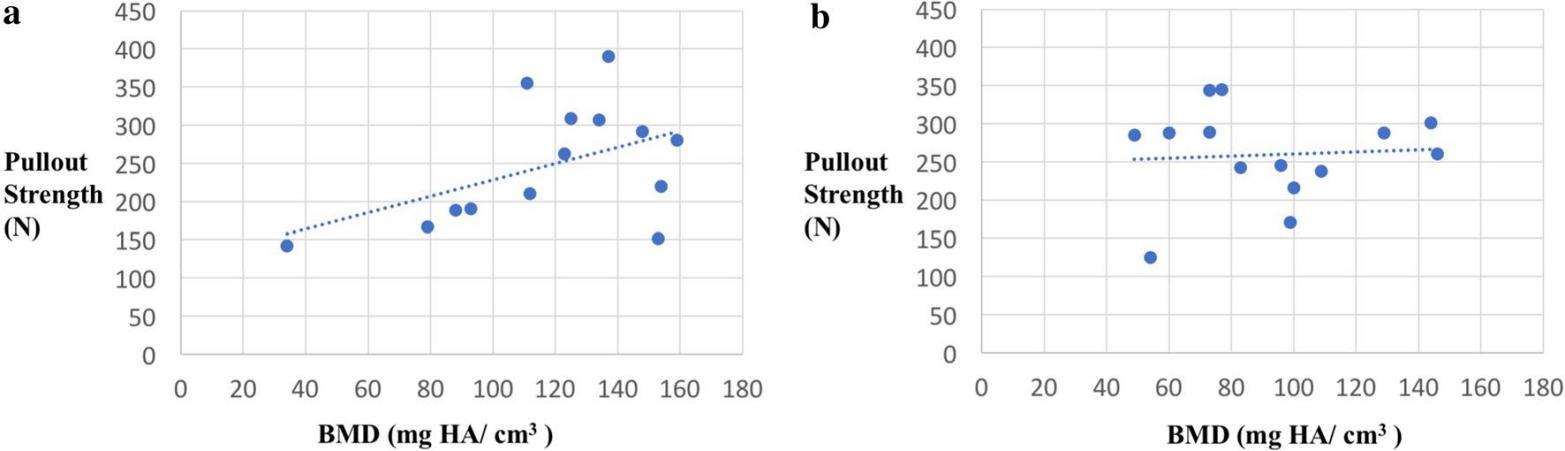
Correlation of **a** all-suture anchor and **b** conventional anchor pullout strength to bone mass density

Analysing a potential correlation between cortical thickness and maximum pullout strength showed a strong linear correlation in all-suture anchors with at least 0.4 mm needed to withstand 200 N, whereas no correlation could be seen in conventional anchors. Regarding local BMD, no correlation could be detected in either the ASA or the conventional anchors (Figs. 5, 6, Table 2).

**Table 2:** Pearson correlation coefficient (*r*) and associated *p* values of cortical thickness and bone mass density in each anchor type

	All-suture anchor	Conventional anchor
*r* cortical thickness	0.82	− 0.22
*p* value	< 0.001^a^	n.s
*r* local BMD	0.48	0.08
*p* value	n.s.	n.s.

^a^Indicates statistical significance

The morphological damage to the bone caused by pullout in terms of volume of the pullout cavity (Fig. 3, Table 1) was significantly smaller for ASA compared to CA (141 mm^3^ vs. 212 mm^3^; *p* = 0.009, disregarding the angle). The pullout cavity ranged from 119 mm^3^ to 173 mm^3^ (ASA) and 182 mm^3^ to 232 mm^3^ (CA) when the anchor was placed at an angle of 45°. Anchor insertion at 90° (CA) and 110° (ASA) resulted in increased bone loss after anchor failure (Table 1). The volume of the bone cavity in CA ranged from 129–460 mm^3^ (90°) and 116–277 mm^3^ (110°) and in ASA from 98–145 mm^3^ (90°) and 110–224 mm^3^ (110°). The two-factor model yielded no significant influence of the implantation angle (n.s., power 0.068) and no significant interaction (n.s., power 0.278), whereas the differences in anchor type showed a significantly larger pullout cavity for CA (*p* = 0.015).

## Discussion

The most important findings of the present study were that the ASA maximum pullout force depends on the thickness of the humeral cortex and that the pullout hole and cortical defect are significantly smaller compared to those with conventional anchors.

Compared to CA, ASA implantation establishes an alternative anchor–bone fixation by deploying the anchor against the inner cortical bone. Several studies have compared the biomechanical properties of conventional and all-suture anchors, but the destruction of the cortical and cancellous bone after implant failure was not evaluated, even though pullout is occasionally observed in clinical practice and might be a severe complication. Reached mean pullout forces for ASA, tested in rotator cuff repairs of human humeral heads, range between 104 and 618 N, and associated bone quality is thought to be a deciding factor [5, 12, 20, 23, 27]. A careful differentiation regarding the cortical and cancellous/trabecular architecture and its individual influence has not yet been performed. Ruder et al. showed that greater tuberosity decortication (1.7 mm) as well as a deeper anchor insertion significantly decreases the ultimate load to failure of an all-suture anchor [26, 27]. According to the results of this study, the ASA maximum pullout force significantly correlates with the thickness of the humeral cortex. In vivo, suture anchors need to withstand a previously estimated maximum force of 200–300 N [2]. The present study shows that this can be achieved by a minimum cortical thickness of 0.4 mm (Fig. 5). Therefore, the results support the hypothesis by Ruder et al. and highlight the importance of cortical thickness as a determining factor when it comes to the endurance of all-suture anchor implantation. So far, the optimal suture anchor insertion site is mostly defined by studies mapping the humeral head, including the greater tubercle according to the BMD [28, 30, 31]. Based on the findings of this study, in the case of ASA implantation, it might be useful to analyse the humeral head regarding the cortical thickness to identify the optimal insertion side. A recent study by Majed et al. demonstrated that the cortical thickness of the proximal humerus ranges between 0.33 and 3.5 mm, with the greater tubercle representing the thinnest part. This is in accordance to the results of the current study, revealing a mean cortical thickness of 0.61 ± 0.26 mm in the proximal anterior part of the greater tubercle 1 cm posterior of the bicipital groove. Therefore, the ASA implantation site should be carefully selected.

Focusing on the BMD, the present results exhibited that there is no correlation between the pullout force and the cancellous BMD measured at the implantation site. Based on the results of this study, cancellous BMD does not seem to be a deciding factor in anchor pullout. This is supported by Bernardoni et al. who also did not observe any association between ASA pullout force and BMD [5]. In contrast, Oh et al. recently published a contrasting view, showing higher ASA pullout strength in higher density bones, although this was in a synthetic bone model [23]. Also regarding CA, the influence of BMD and cortical thickness on anchor pullout is highly controversial with differing results. In this context, the results of this study support the opinion that suture anchor pullout seems to be influenced by other factors such as trabecular microstructure and its interactions to different anchor types. These factors are not reflected by BMD measurements. Moreover, it needs to be noted that the differing results might also be explained by the different methods used to determine the BMD (DXA, CT and micro-CT), which reduces comparability of the different studies. Thus, suture anchor failure seems to be influenced by multiple factors [1, 9, 28, 31].

Furthermore, the optimal angle of anchor insertion remains up for discussion. In 1995, Burkhart first proposed the so-called “deadman’s” angle to be the best choice based on trigonometric calculations [7]. Since then, the optimal insertion angle has been widely discussed in several biomechanical models and with different anchor types [8, 14, 17, 22, 23, 29]. In contrast to Burkhart, other authors have demonstrated superior biomechanical properties at an angle of 90° or more [8, 13, 22, 29]. This study shows that, in the case of anchor pullout, the cortical destruction is higher if the anchor, regardless of type, is inserted at a 45° angle. Considering potential revision surgery, it might, therefore, not be recommendable. A previously described failure mode of conventional anchors placed at the deadman’s angle has been the so-called wind shield wiper effect. If this was the case though, one would expect more pronounced bone loss at an angle of 45° which could not be proven by this study. Furthermore, the morphometric 3D imaging of the bone cavity questions the existence of that effect due to its inconsolable morphology [8]. Overall, according to the results of this study, placing either the ASA or the CA at a 45° angle seems to be unfavourable while the specific reason for this observation remains open for debate. Anchor failure displayed by anchor pullout can be a devastating complication, and challenges every arthroscopic surgeon. In this context, ASA are thought to be superior to conventional anchors because of their smaller footprint and through less bone removal during implantation. The results of this study provide evidence that, in the case of anchor pullout, ASA show a significantly smaller cortical defect compared to conventional anchor [3, 10, 11, 19, 21]. Moreover, this study confirms that the volume of the bone cavity induced by ASA pullout compared to conventional anchors is significantly smaller. Due to this bone-preserving ability, ASA implantation might provide advantages in case of revision surgery. Still, the long-term biological effects of ASA on the bone, such as the collection of perianchor fluid or the development of bone cysts, need to be reviewed [16].

The limitations of this study include the in vitro setup. Direct clinical translation is, therefore, limited. Furthermore, the availability of human humeral heads for in vitro testing is restricted and this limitation in sample size also limits the power to accept the null hypotheses.

The determining variety in morphology, especially of ASA, which induce the observed differences in bone damage, is not yet known. Since only one ASA was investigated, no general conclusion for this anchor type may be made.

## Conclusion

The present study demonstrates that the pullout force of all-suture anchors, in contrast to conventional anchors, depends on the thickness of the humeral cortex. Furthermore, following anchor pullout, ASA show a reduced cortical defect as well as a smaller bone cavity.

With regard to the clinical implications, all-suture anchors are advantageous due to their bone-preserving ability enabling additional points of fixation within the same region, as well as maintaining bone substrate in case of operative revision. Attention should be paid to the fact that all-suture anchors are best placed at regions with pronounced cortical thickness. Thus, intraoperative decortication should not be performed in the direct area of anchor placement and the cortex should be carefully evaluated in preoperative imaging.
